# Supplementary material for: The interactome of intact mitochondria by cross-linking mass spectrometry provides evidence for coexisting respiratory supercomplexes
Source: Mol Cell Proteomics. 2017 Dec 8;17(2):216–32. doi: 10.1074/mcp.RA117.000470 (PMC5795388; doi:10.1074/mcp.RA117.000470)
Supplement: Supplemental Data [file supp_RA117.000470_133922_0_supp_23978_fzffwf.zip › spectra_annotation/mito_DR_spectra_annotation/56-1-3-1-7-1.pdf]

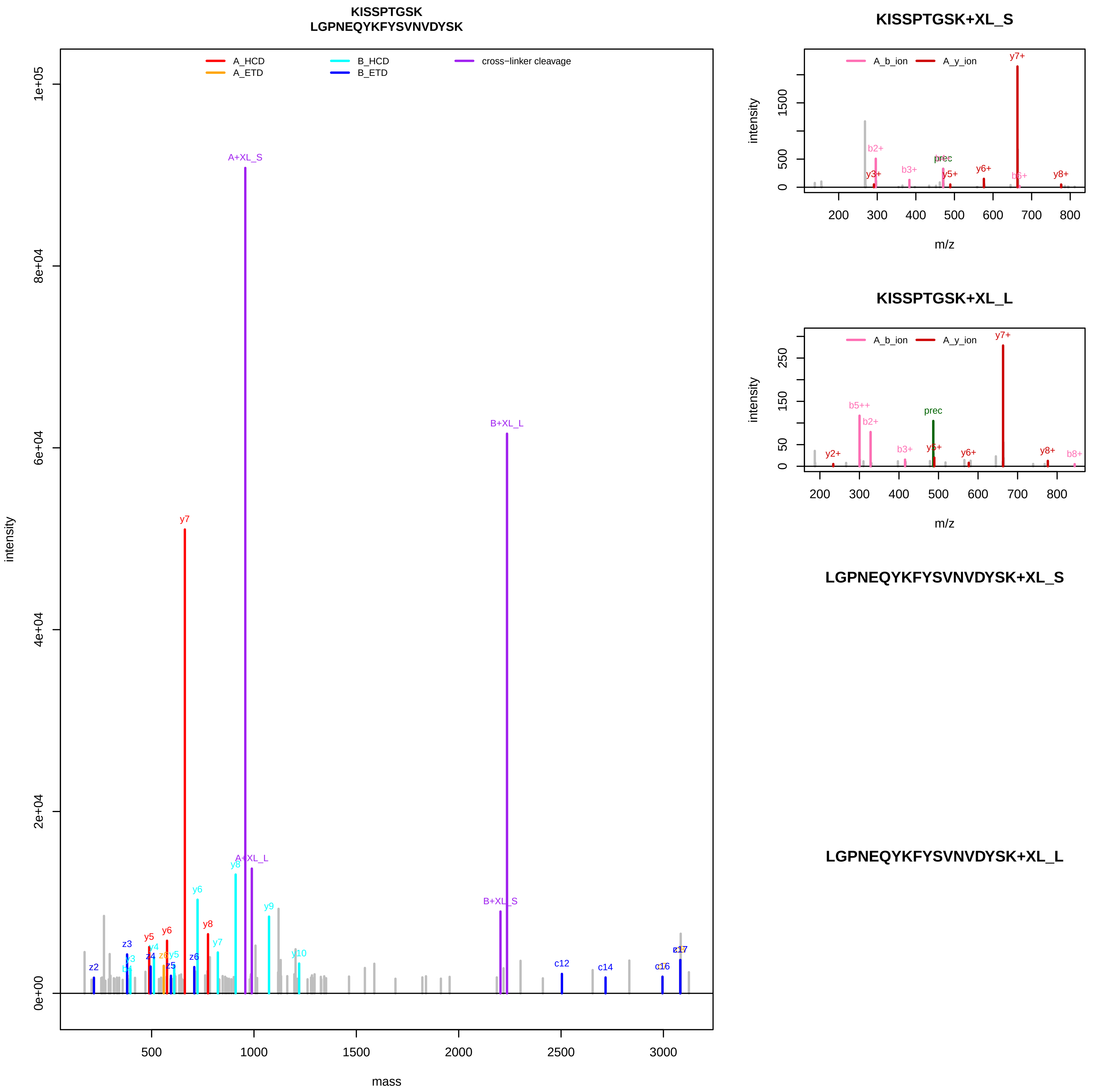
KISSPTGSK
LGPNEQYKFYSVNVDYSK
A_HCD
A_ETD
B_HCD
B_ETD
cross−linker cleavage
A+XL_S
B+XL_L
A+XL_L
B+XL_S
y5
y6
y7
y8
y3
y4
y9
y10
z2
z3
z4
z5
z6
c12
c14
c16
c17
intensity
mass
KISSPTGSK+XL_S
A_b_ion
A_y_ion
b2+
b3+
y3+
y5+
y6+
y7+
y8+
prec
m/z
KISSPTGSK+XL_L
b5++
y2+
b8+
LGPNEQYKFYSVNVDYSK+XL_S
LGPNEQYKFYSVNVDYSK+XL_L
